# RT-LAMP Multicenter Study for SARS-CoV-2 Genome Molecular Detection in Brazilian Swab and Saliva Samples

**DOI:** 10.3390/diagnostics13020210

**Published:** 2023-01-06

**Authors:** Vanessa Duarte da Costa, Alanna Calheiros Santos, Lucas Lima da Silva, Wilian Jean Wiggers, Claudia Alexandra Pontes Ivantes, Danielle Malta Lima, Jeová Keny Baima Colares, Deusilene Souza Vieira Dallacqua, Ana Rita Coimbra Motta-Castro, Alberto Martín Rivera Dávila, Sheila Araujo Teles, Megmar Aparecida dos Santos Carneiro, Karlla Antonieta Amorim Caetano, Fernando Antonio Costa Anunciação, Vanessa Salete de Paula, Livia Melo Villar, on behalf of The Brazilian COVID-19 Research Group

**Affiliations:** 1 Brazilian Reference Laboratory of Viral Hepatitis, Oswaldo Cruz Institute, FIOCRUZ, Rio de Janeiro 21040-360, Brazil; 2 Service of Gastroenterology, Hepatology and Liver Transplantation, Hospital Nossa Senhora das Graças, Curitiba 80810-040, Brazil; 3 Graduate Program in Medical Sciences, University of Fortaleza, Fortaleza 60811-905, Brazil; 4 Molecular Virology Laboratory, Oswaldo Cruz Foundation, FIOCRUZ, Porto Velho 76812-245, Brazil; 5 Federal Faculty, University of Mato Grosso do Sul, Campo Grande 79070-900, Brazil; 6 Computational and Systems Biology Laboratory, Graduate Program in Biodiversity and Health, Oswaldo Cruz Institute, FIOCRUZ, Rio de Janeiro 21040-360, Brazil; 7 Federal Faculty, University of Goiás, Goiânia 74690-900, Brazil; 8 Hospital Getúlio Vargas, Teresina 64001-020, Brazil; 9 Natan Portela Institute of Tropical Diseases, Teresina 64002-510, Brazil; 10 Molecular Virology Laboratory, Oswaldo Cruz Institute, FIOCRUZ, Rio de Janeiro 21040-360, Brazil

**Keywords:** LAMP, SARS-CoV-2, COVID-19, diagnosis

## Abstract

Reverse transcription loop-mediated isothermal amplification (RT-LAMP) is a rapid method that can replace RT-qPCR. A simple molecular assay for SARS-CoV-2 RNA detection in gold-standard diagnosis through swabs and alternative specimens such as saliva could be helpful in promoting genomic surveillance. A multicenter study was conducted to evaluate the RT-LAMP assay method as an alternative for the molecular detection of SARS-CoV-2 lineages in swab and saliva samples. A total of 350 swabs from individuals with (*n* = 276) or without (*n* = 74) COVID-19 tested by RT-qPCR were collected. Paired saliva was also collected from 90 individuals who had SARS-CoV-2 RNA that was detectable (*n* = 30) or undetectable (*n* = 60) via RT-qPCR. For the RT-LAMP methodology, six primers were used for ORF1 gene amplification. As for SARS-CoV-2 genotyping, 39 swabs had the whole genome sequenced by MinION. The sensitivity of RT-LAMP to the swab was 90.2%. For the swab samples with Ct ≤ 30, the sensitivity improved by 96%. Considering saliva with Ct ≤ 30 in RT-qPCR testing, the RT-LAMP sensitivity was 100%. The RT-LAMP specificity was 100% for both the swab and saliva samples. This RT-LAMP assay was capable of detecting all the SARS-CoV-2 lineages circulating in the Brazilian swab samples. The RT-LAMP method has significant potential for use in clinical routines since it was capable of detecting SARS-CoV-2 RNA in swab and saliva samples.

## 1. Introduction

The coronavirus disease 2019 (COVID-19) pandemic caused by the severe acute respiratory syndrome coronavirus 2 (SARS-CoV-2) represented a global public health concern with more than 6 million deaths [1,2,3]. Diagnostic testing for COVID-19 has a critical role for epidemiological surveillance and consequently helps health professionals and authorities to take preventive or clinical measures. Currently, the gold standard of COVID-19 diagnosis is made through naso- and oropharyngeal swabs [4]. Since the collection of these respiratory samples is considered invasive, saliva is considered an alternative specimen for COVID-19 diagnosis since it has advantages such as a minor risk of transmission, being less invasive, the option of self-collection, a simple procedure, and the possibility of developing point-of-care tests [5,6,7].

According to Centers for Disease Control and Prevention (CDC), quantitative reverse transcription polymerase chain reaction (RT-qPCR) assays represent the standard method to detect current infections of SARS-CoV-2 [4,8]. Previous studies have demonstrated that a RT-qPCR cycle threshold (Ct) < 30 could assume a higher correlation with infectivity based on antigen test sensitivity and virus culture [9,10,11,12]. Despite the fact that PCR-based assays can provide results relatively fast, these techniques require capable professionals, specific equipment for precise temperature cycling, and adequate laboratory infrastructure. In addition, these conditions cannot be accessed by countries with limited resources or in places with geographical conditions that are difficult to access, such as those observed in several regions of the world. Given these unsatisfactory conditions, other nucleic acid amplification methods should be developed for the accurate diagnosis of SARS-CoV-2. Notomi et al. (2000) [13] developed a single-point temperature technique named loop-mediated isothermal amplification (LAMP), which combines advantages such as the simple visualization of amplification products, fast procedures, no necessity of a real-time thermal cycler, simple laboratory resources, and basic personnel training [14].

Considering its simplicity and given the importance of developing and optimizing an alternative protocol for SARS-CoV-2 genome detection compared to the gold standard of RT-qPCR, reverse transcription-LAMP (RT-LAMP) assays have been used by many laboratories for COVID-19 diagnosis using respiratory swabs and saliva [15,16,17]. The referred method has disadvantages, such as a requirement for the design of six primers and a restricted availability of reagents and equipment in some countries; however, its fast and cost-effective procedures justify the necessity of an alternative to RT-qPCR. Up to November 11st, 2022, Brazil had more than 34 million cases and 688,487 confirmed deaths, which justifies the necessity for more studies in our country for the development of rapid diagnostic tests in the context of the COVID-19 pandemic [3]. In addition, the knowledge and control of SARS-CoV-2 lineages, mainly the Brazilian variants gamma (Manaus) and zeta (Rio de Janeiro), derived from the B.1.1.28 lineage, and the variants delta and omicron, first identified at India and South Africa, respectively, is essential to promote genomic surveillance through diagnostic tests. In this study, we aimed to evaluate the RT-LAMP assay method as an alternative for the molecular detection of SARS-CoV-2 lineages in swab and saliva samples originating from all Brazilian geographic regions.

## 2. Materials and Methods

### 2.1. Samples Description

#### 2.1.1. Naso- and Oropharyngeal Swab Samples

A total of 350 respiratory samples (naso- and oropharyngeal) were collected, with swabs and temporarily stored in 0.9% saline until final storage at −70 °C. A convenience sampling of swabs based on the regional positivity of SARS-CoV-2 was collected between May 2020 and August 2021 from symptomatic or asymptomatic (close contact with positive individuals for COVID-19) participants attending ambulatories (*n* = 235), hospitals (*n* = 14) and from those screened in the community (*n* = 101) from all Brazilian geographic regions. Of the 350 individuals, 276 had detectable SARS-CoV-2 RNA via swab RT-qPCR performed as described later, and 74 had undetectable SARS-CoV-2 RNA (Table 1).

#### 2.1.2. Saliva Samples

Saliva collection was carried out via RT-LAMP swabs from 90 subjects with or without COVID-19 that were previously tested using a commercial collector (Salivette, Sarstedt) between July 2020 and August 2021. SARS-CoV-2 RNA was detectable in saliva via RT-qPCR performed as described later for 30 participants and SARS-CoV-2 RNA was undetectable for 60 participants (Table 2).

### 2.2. Ethical Approval

Samples were collected after each patient had signed informed consent. This study was approved by the Brazilian Ethics Committee (CONEP under CAAE 30468620.5.0000.5248).

### 2.3. Viral RNA Extraction

SARS-CoV-2 RNA was extracted from 140 µL of naso- or oropharyngeal swabs and saliva samples by commercial kit QIAamp Viral RNA Mini Kit (QIAGEN, Hilden, Germany) according to the manufacturer’s instructions.

### 2.4. SARS-CoV-2 TaqMan RT-qPCR Assay

RT-qPCR amplification assays for swabs and saliva were carried out using reagents from AgPath-ID™ One-Step RT-PCR (Thermo Fisher Scientific, Waltham, Massachusetts, USA). A set of probe-associated primers (assay) aimed at SARS-CoV-2 nucleocapsid (N1 and N2) and envelope (E) genes was used [18,19]. Endogenous control Ribonuclease P/MRP Subunit P30 (RPP30) was also included into the assay to assess sample integrity. The RT-qPCR mixture contained 10 µM of sense and antisense primers, 5 µM of probe, 2X RT-PCR buffer, 25X RT-PCR Enzyme mix (ArrayScript™ Reverse Transcriptase and AmpliTaq Gold^®^ polymerase), and 5 µL of viral RNA extracted from swabs and 7 µL from saliva. The conditions for the RT-qPCR at Rotor-Gene Q equipment (QIAGEN) were as follows: 45 °C for 15 min for reverse transcription followed by enzyme activation at 95 °C for 10 min, and then 45 cycles were conducted at 95 °C for 15 s and at 55 °C for 45 s. Ct values below 40 for two of the three genes represented positive results. Fluorescence readings were performed using the FAM channel, and the analysis of the results was performed using the Rotor-gene Q series program with the threshold set at 0.1.

### 2.5. Swab and Saliva RT-LAMP Assay

The RT-LAMP primer sets used in this study were designed by Lamb et al. (2020) [20] against the nonstructural protein 3 (NSP3) coding region of ORF1ab. The RT-LAMP reaction for swab and saliva was conducted in a total volume of 25 μL of 1X isothermal amplification buffer, 1.4 mM deoxynucleoside triphosphates (dNTPs), 6 mM MgSO4, 1.6 µM FIP/BIP, 0.2 µM F3/B3, 0.4 µM Loop F/B primers, 0.32 U/µL Bst 2 WarmStart DNA polymerase (New England Biolabs, Ipswich, Massachusetts, USA), 15 U/µL WarmStart RTx Reverse Transcriptase (New England Biolabs), 8 µL of nuclease-free water, and 2 µL of viral RNA. A no-template control (NTC) with the same water used for the reaction contained substituted viral RNA. The reaction mixtures were incubated using a PCR thermocycler at 63 °C for 45 min for the swab samples and at 65 °C for 60 min for the saliva samples followed by enzyme inactivation at 80 °C for 10 min. Amplification products were fractionated by 3% agarose gel electrophoresis, stained with fluorescent dye ethidium bromide, and visualized under ultraviolet (UV) light.

### 2.6. SARS-CoV-2 Whole-Genome Genotyping from Swab Samples

After RT-LAMP amplification, 39 swabs with RT-qPCR N1 Ct ≤ 30 were selected to have their SARS-CoV-2 lineage genotyped. Initially, a SuperScript™ IV First-Strand Synthesis System (Thermo Fisher Scientific) was used for cDNA synthesis. In a total volume of 13 μL, first-strand cDNA was produced using 50 ng/μL of random hexamer, 10 mM of dNTP mix, and 11 μL of template RNA followed by incubation at 65 °C for 5 min and cooling on ice. For totalizing to 20 μL, a mixture of 5X SuperScript IV Buffer, 100 mM DTT, ribonuclease inhibitor (40 U/μL), and SuperScript^®^ IV Reverse Transcriptase (200 U/μL) was added to the first step. This reaction was incubated at 42 °C for 50 min and at 70 °C for 10 min.

Next, two separate pools of primers (ARTIC nCoV-2019 V3 panel, see https://artic.network/resources/ncov/ncov-amplicon-v3.pdf (accessed on 24 November 2022)) were used for amplification with Q5 Hot Start High-Fidelity DNA Polymerase (New England Biolabs). A reaction with a final volume of 25 μL was conducted with the following reagents: 5X Q5 Reaction Buffer, 10 mM of dNTP, 10 μM of each pool of primers, and Q5 Hot Start DNA Polymerase (0.02 U/µL) with the volume adjusted with nuclease-free water. For this amplification, 2.5 μL of cDNA was added. The thermocycling conditions were 98 °C for 30 s for the initial activation of DNA polymerase followed by 40 cycles at 98 °C for 15 s, 65 °C for 5 min, and 72 °C for 10 s and a final elongation at 72 °C for 10 min.

PCR amplicon pools were diluted (2.5 μL of each pool with 45 μL of nuclease-free water totalizing to 50 μL) and submitted to end-repair and dA-tailing with an NEBNext Ultra II End Repair/dA tailing module (New England Biolabs). A final volume of 10 μL remained at room temperature for 15 min, was incubated at 65 °C for 15 min, and was cooled on ice for 1 min. For native barcode ligation, end prep products (0.75 μL) were added to a mixture with native barcodes (EXP-NBD104 and EXP-NBD114, Oxford Nanopore Technologies, Oxford, UK) and Blunt/TA Ligase master mix (New England Biolabs). The mix was incubated at room temperature for 20 min, incubated at 65 °C for 10 min, and cooled on ice for 1 min. Following this, all the pooled barcoded libraries were purified using a ProNex^®^ Size-Selective Purification System (Promega, Madison, WI, USA), quantified by a fluorometer Qubit dsDNA HS Assay Kit (Thermo Fisher Scientific), and used for adaptor insertion with an NEBNext Quick Ligation Module (New England Biolabs). A new purification with ProNex magnetic beads and short fragment buffer (SFB) and elution buffer (EB) reagents from a Ligation Sequencing Kit (SQK-LSK109, Oxford Nanopore Technologies) resulted in 15 μL of the eluted sequencing library. Library quantification was conducted using a Qubit dsDNA HS Assay Kit (Thermo Fisher Scientific), and 15 ng of DNA was selected to be the minimum necessary amount of DNA to reach the maximum run yield. Following this, a primed R9.4.1 flow cell (FLO-MIN106D) was used to load the library and was sequenced on a MinION Mk1B device. The quality assessment of the generated reads, assembly, and alignment with reference genomes were carried out through the Galaxy platform.

### 2.7. Data Analysis

Descriptive statistical analysis was performed with the calculation of means and standard deviation. Statistical analysis was determined using GraphPad InStat 3 (GraphPad InStat Software, San Diego, CA, USA).

SARS-CoV-2 RNA detection by RT-qPCR in the swab samples was used as the gold standard method for the assessment of sensitivity, specificity, positive predictive value (PPV), and negative predictive value (NPV). Respective 95% confidence intervals (95% CIs) were calculated by the modified Wald method. SARS-CoV-2 detection in the saliva samples using RT-qPCR was the gold standard for determining the sensitivity and specificity of the LAMP method in the saliva samples.

To evaluate the effectiveness of the RT-LAMP method with the swab samples for detecting SARS-CoV-2 RNA, we measured the precision and reproducibility from a 1:10 serial dilution of a selected sample with an N1 Ct value of 17 (3 concentrations referring to 10^4^, 10^2^, and 10 copies/μL were selected). For precision, 10 replicates of each concentration were evaluated on the same day by the same operator. As for reproducibility, 10 replicates were similarly tested on two consecutive days by the same operator. Referring to the effectiveness of the RT-LAMP method for the saliva samples, a selected swab sample with an N1 Ct value of 17 was added into a pool of negative saliva and a 1:10 serial dilution was conducted. Five dilutions (10^1^ to 10^5^) were tested using the RT-LAMP method for the saliva samples.

## 3. Results

### 3.1. Molecular Assays for Nasopharyngeal Swabs and Saliva Samples

In this study, a total of 350 individuals had nasopharyngeal swab samples collected (Table 3). The swab samples from 276 patients had detectable SARS-CoV-2 RNA using RT-qPCR (Table 4). Considering the SARS-CoV-2 N1 target, the mean Ct was 24 ± 4.9.

Regarding RT-LAMP for the positive swab samples previously tested by RT-qPCR, an overall sensitivity of 90.2% was identified, i.e., 249/276 SARS-CoV-2 RNA were detected and 27/276 (9.8%) were undetectable. However, for the swab samples with Ct ≤ 30 for the SARS-CoV-2 N1 target region (*n* = 249), the sensitivity improved to 96%, of which 239/249 had a positive result. Ten negative samples with Ct ≤ 30 had a mean Ct of 26.3 ± 1.81. A specificity of 100% (74/74) was observed since all the negative swabs tested by RT-qPCR also had a negative result using RT-LAMP.

**Figure 1:**
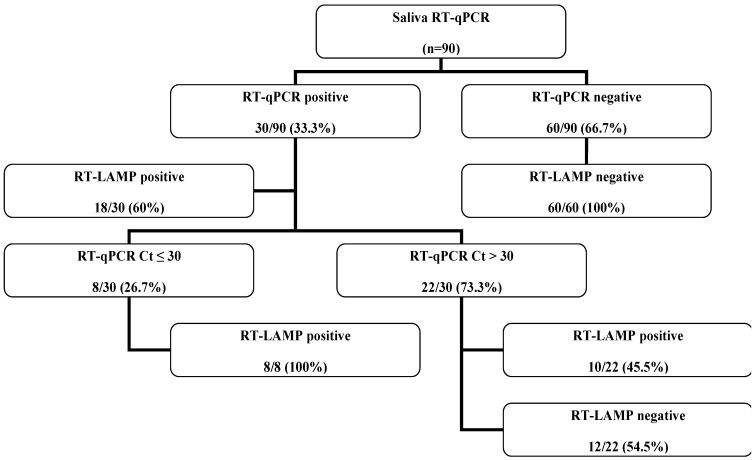
Flowchart demonstrating sensitivity and specificity results for saliva molecular assays.

Given that saliva samples were not available from some ambulatories, 90 saliva samples tested by in-house RT-qPCR were selected for molecular analysis (Figure 1). The results from RT-qPCR determined that 30/90 (33.3%) were positive and 60/90 (66.7%) were negative. Overall, the sensitivity of RT-LAMP for the saliva samples was 60% (18/30). However, for the saliva samples with Ct ≤ 30 using RT-qPCR (*n* = 8), the sensitivity of RT-LAMP was 100% (8/8). A specificity of 100% (60/60) was identified since all the negative saliva samples identified by RT-qPCR were also negative using RT-LAMP.

### 3.2. Comparison between Swab Collection Period and RT-LAMP Results

The period between the onset of symptoms or contact with an individual with positive COVID-19 and swab collection was assessed via questionnaire for 118 patients from Rio de Janeiro and Mato Grosso do Sul. In order to evaluate if the time of swab collection might have influenced the RT-LAMP results, three periods were defined (Table 5).

### 3.3. SARS-CoV-2 Genotyping via MinION

**Figure 2:**
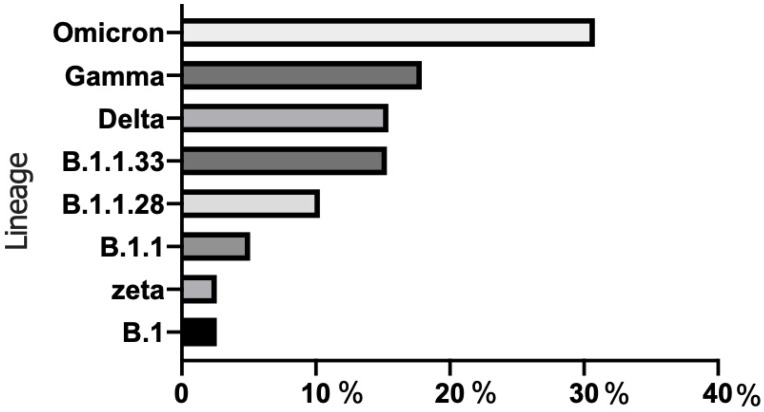
Genotyping and prevalence of SARS-CoV-2 variants in swab samples.

A total of 39 swab samples with N1 Ct ≤ 30 using RT-qPCR were submitted to high-throughput sequencing via a MinION device to determine if the RT-LAMP method was capable of detecting different SARS-CoV-2 lineages. The mean N1 Ct value was 23.2 ± 3.7. The SARS-CoV-2 lineages identified were B.1 (1/39; 2.6%), B.1.1 (2/39; 5.1%), B.1.1.28 (4/39; 10.3%), B.1.1.33 (6/39; 15.3%), gamma (7/39; 17.9%), zeta (1/39; 2.6%), delta (6/39; 15.4%), and omicron BA.1 (12/39; 30.8%) (Figure 2). RT-LAMP was able to detect SARS-CoV-2 RNA for 34/35 of the swab samples (97.1%). Only one sample from a patient infected by SARS-CoV-2 lineage B.1.1 was not detected by RT-LAMP.

### 3.4. Data Analysis

The precision of the RT-LAMP method with the swab samples was evaluated after selecting three concentrations (10^4^, 10^2^, and 10 copies/μL) from a 1:10 serial dilution of a clinical swab (RT-qPCR N1 Ct = 17). For the concentration of 10^4^ copies/μL, all ten replicates (100%; 10/10) had an amplification at 3% agarose gel electrophoresis. As for the concentrations of 10^2^ and 10 copies/μL, 9/10 (90%) and 2/10 (20%) were positive, respectively.

As for reproducibility, ten replicates of each concentration were similarly tested on two consecutive days by the same operator. Even with the limitation of a low replicate number, for the concentration of 10^4^ copies/μL, a 100% reproducibility was observed since all ten replicates (100%; 10/10) had an amplification at 3% agarose gel electrophoresis over both days. Considering the concentration of 10^2^ copies/μL, an 80% reproducibility was identified, where only 2 of the 10 replicates had different results on both days. As for the concentration of 10 copies/μL, 10 replicates with no amplification at 3% agarose gel electrophoresis on the first day had the same result on the second day, corresponding to a reproducibility of 100%.

Referring to the RT-LAMP results for the saliva samples from a 1:10 serial dilution, the results of 3% agarose gel electrophoresis indicated an amplification of four out of five dilutions (10^1^ to 10^4^) with a detection limit of 1 copy/μL.

## 4. Discussion

Several factors influence the molecular detection of SARS-CoV-2 including viral loads, the onset of symptoms, the ease of collection for an inpatient or outpatient, the handling of clinical specimens, and appropriate RNA extraction [21,22]. Indeed, various RT-LAMP molecular assays for SARS-CoV-2 infection diagnosis have been developed with a broad range of sensitivity and specificity rates [11,12,23,24,25]. In reference to the sensitivity for different locations, a variation was found for Paraná (80% for swabs Ct ≤ 30 and 55.6% for swabs Ct >30). This contrast might have been associated with a more difficult access and collection in patients that were hospitalized, which might have resulted in a possible lower viral load if the nasopharyngeal swabs were not well positioned as per the standard procedure. The present study identified an overall sensitivity of 90.2% (249/276) for RT-LAMP swab analysis, similar to the sensitivity of 91.45% (117/124) observed by Haq and collaborators (2021) [25]. For positive swabs with Ct ≤ 30 (*n* = 249), which indicates a high viral load, sensitivities from all Brazilian regions, except Paraná, were higher than 90% (239/249), corroborating another study from Kundrod et al. (2022) [26], who observed a positive agreement of 100% with RT-LAMP for samples with Ct < 30 and 69–91% for samples with Ct < 40. Previous studies have also observed a comparison between RT-qPCR Ct values and RT-LAMP positivity. The absence of RT-qPCR standardization between researchers and the limited information about residual SARS-CoV-2 RNA make it difficult to determine which Ct value should be considered for transmissibility. Due to the limited data, alternative methodologies should be developed to improve SARS-CoV-2 molecular diagnosis, even for low viral loads. Iqbal and collaborators (2022) [23] have observed a totality of 206 out of 240 SARS-CoV-2-positive samples up to Ct ≤ 40 that were tested positive by LAMP, representing a sensitivity of 85.8%. Referring to 80 SARS-CoV-2-negative samples that were tested negative by LAMP, a previous study had a specificity of 100%, which corroborated with the present research, since all the 74 SARS-CoV-2 negative swabs tested via RT-qPCR were also negative using RT-LAMP. A previous Canadian study from Lu et al. (2022) [24] evaluated 30 positive and 36 negative nasopharyngeal samples tested via RT-qPCR and identified a sensitivity and specificity of 90% and 100%, respectively. Out of the 3/30 positive samples that were not detectable by RT-LAMP, all had Ct values of 36 and 37, corresponding to low copy levels. The swab samples from Rio de Janeiro and Mato Grosso do Sul detailed the period between collection and RT-qPCR testing. Based on these data, the importance of testing swab samples until ten days after the onset of symptoms represented an important parameter for RT-LAMP positivity. A systematic review of longitudinal studies of RT-PCR test results in symptomatic SARS-CoV-2 individuals has indicated that the highest proportion of virus detection was from nasopharyngeal swabs collected between 0 and 4 days post symptom onset at 89%, dropping to 54% after 10 to 14 days [27]. A previous study from Inaba M et al. (2021) [28] described that up to the 9th day after onset, RT-LAMP had a positivity of 92.8%, and the sensitivity and specificity compared with RT-qPCR was 100%. In the present study, RT-LAMP was capable of detecting SARS-CoV-2 RNA in 98.2% (110/112) of the swab samples collected up to 10 days after symptom onset. Our limitation was due to the number of samples collected after 10 days post symptoms (*n* = 6). Even with this limitation, both studies indicated a decreased positivity for samples collected after the 10th day after onset (25% for the previous study and 50% for the present research).

The approach of using saliva as a potential alternative to replace nasopharyngeal swabs for the molecular detection of SARS-CoV-2 has been discussed, since it is considered an easy-to-obtain sample, represents a non-invasive collection method, and has the option of self-collection [29,30,31,32,33]. ACE2 expression in the epithelial cells of the salivary gland has been confirmed, and this makes them predisposed to be infected by SARS-CoV-2, even if in a minor way compared to other tissues such as the gastrointestinal system and renal and cardiac muscles [34,35]. Saliva is a biological fluid comprising different components such as electrolytes and proteins, represented by enzymes, cytokines, and hormones [36]. Given this composition, the probability of viral RNA degradation is significant [37]. A previous study from To and collaborators (2020) [38] quantified the SARS-CoV-2 viral load of 23 patients with COVID-19, and 87% had SARS-CoV-2 RNA detectable in their saliva. In addition, the presence of viral RNA was observed in these same samples for more than twenty days in seven patients, from which the authors suggested the use of saliva for the initial diagnosis of COVID-19.

An overall sensitivity of 60% (18/30) was identified in the present study for RT-LAMP with saliva samples. This low percentage might have been associated with the high RT-qPCR Ct value (Ct ≥ 32) for 12 saliva samples that ended up negative using RT-LAMP. A sensitivity of 100% was confirmed for eight saliva samples with Ct ≤ 30, demonstrating the importance of a high viral load for RT-LAMP analysis. It is important to highlight that 10 saliva samples with Ct > 30 were positive using RT-LAMP. This might be associated with a reduced period between saliva collection, viral RNA extraction and RT-LAMP testing, and/or viral subpopulations with a low sequence diversity in the primer binding. A previous study related to saliva analysis indicated a 77.2% overall sensitivity and a 97% specificity [39]. However, a sensitivity of 93.2% was found for saliva containing at least 10^2^ viral copies/µL. Our study had the limitation of not being able to quantify the viral RNA extracted from saliva; however, a 1:10 serial dilution from saliva with a high viral load (Ct = 17) was made, and a detection limit of 1 copy/μL was achieved. As we also mentioned, Kundrod et al. (2022) [26] discussed the influence of Ct values in RT-LAMP results. For individuals with a nasopharyngeal swab with Ct < 30, the RT-LAMP sensitivity for both saliva and nasal swabs was approximately 60%, a result that was similar to that found in the present study. In-house RT-LAMP is described as a molecular assay that may be conducted in up to 60 min. This advantage could be compared to commercial methods such as the chemiluminescent enzyme immunoassay Lumipulse G SARS-CoV-2 Ag kit (Fujirebio, Tokyo, Japan) that can detect SARS-CoV-2 antigens in 35 min for nasopharyngeal swabs and saliva. However, there are differences between them since the first one corresponds to an in-house single-point temperature technique for viral RNA detection, while the last commercial one is associated with viral antigen detection.

This RT-LAMP assay was capable of detecting all SARS-CoV-2 lineages circulating in the Brazilian swab samples collected since May 2020. Initially, the test was able to detect lineage B.1.1.28, first described in Brazil in March 2020 [40]. In the middle of 2020, new lineages descended from B.1.1.28, named gamma and zeta, emerged in the Brazilian states of Amazonas and Rio de Janeiro, respectively [41,42]. A study has evaluated the temporal spreading and evolution of SARS-CoV-2 in the beginning of the second pandemic wave in Brazil and highlighted the fast dissemination of these two variants in the last trimester of 2020 to all Brazilian regions [43]. Given these data, the originally detected gamma variant in Manaus was identified in the present study in seven swab samples collected in May 2021 from individuals resident in Rio de Janeiro. The variants of concern delta (B.1.617.2), first detected in India in October 2020 [44], and omicron (B.1.1.529), first detected in specimens collected on November 11th, 2021, in Botswana and on November 14th, 2021, in South Africa [45], were also detected by this RT-LAMP study. Eighteen swab samples collected between September 2021 and January 2022 were genotyped and indicated either the delta (6/39) or omicron (12/39) variant. Given the large number of mutations in the spike gene, primer sets against the ORF1ab region seemed to be an alternative for virus detection. Luo and collaborators (2022) [46] highlighted the necessity of the continuous exploration of RT-LAMP standardization for evaluating emerging SARS-CoV-2 variants and they had success in detecting the genome of SARS-CoV-2 variants in 41 suspected COVID-19 patients. In a previous study, given the better discrimination between SARS-CoV-2 variants and other related viruses, the authors also used a primer set for the conserved binding sites in the ORF1ab gene, which was chosen for an RT-LAMP assay final validation of patient samples [47]. However, RT-LAMP targeting of the SARS-CoV-2 N and E genes has been used for the molecular detection of the SARS-CoV-2 omicron variant in previous Brazilian research by Almeida et al. (2022) [48]. More importantly, the molecular diagnostic test sensitivity is basically related to the conserved binding site for RT-LAMP primers.

## 5. Conclusions

In summary, this RT-LAMP assay was capable of detecting SARS-CoV-2 RNA in swab and saliva samples with high sensitivity and specificity. This study might have had limitations in terms of the sample size compared with other studies; however, the testing samples until ten days after the onset of symptoms and with a high viral load (RT-qPCR Ct ≤ 30) represented important parameters for positivity using RT-LAMP. In addition, our test was able to detect the SARS-CoV-2 lineages circulating in Brazilian samples with high sensitivity (97.4%). In conclusion, RT-LAMP has significant potential for use in clinical routines since it involves fast and cost-effective procedures compared to RT-qPCR, which suggests its use for the screening diagnosis of symptomatic patients in the initial phase of infection.

## Figures and Tables

**Table 1:** Panel of swab samples according to RT-qPCR result.

Panel of Swab Samples	*n*
**1. SARS-CoV-2 RT-qPCR positive swabs**	**276**
*** Swab N1 Ct ≤ 30***	**249**
** North region**	
Rondônia	10
** Northeast region**	
Ceará	52
Piauí	22
** Midwest region**	
Mato Grosso do Sul	101
Goiás	13
** Southeast region**	
Rio de Janeiro	46
** South region**	
Paraná	5
*** Swab N1 Ct >30***	**27**
** Southeast region**	
Rio de Janeiro	18
** South region**	
Paraná	9
**2. SARS-CoV-2 RT-qPCR negative swabs**	**74**
** North region**	
Rondônia	5
** Midwest region**	
Goiás	19
** Southeast region**	
Rio de Janeiro	50

**Table 2:** Panel of saliva samples according to RT-qPCR results.

Panel of Saliva Samples	*n*
**1. SARS-CoV-2 RT-qPCR positive saliva**	**30**
*** Swab N1 Ct ≤ 30***	**8**
** Southeast region**	
Rio de Janeiro	5
** South region**	
Paraná	3
*** Swab N1 Ct >30***	**22**
** Southeast region**	
Rio de Janeiro	20
** South region**	
Paraná	2
**2. SARS-CoV-2 RT-qPCR negative saliva**	**60**
** Midwest region**	
Goiás	38
** Southeast region**	
Rio de Janeiro	19
** South region**	
Paraná	3

**Table 3:** Variable analysis for comparison between all swabs tested by RT-qPCR and RT-LAMP.

Variable Analyzed	Molecular Tests Comparison
	RT-qPCR vs. RT-LAMP (*n* = 350)
Sensitivity % (CI 95%)	77 (72.19–81.35)
Specificity % (CI 95%)	100 (94.09–100)
True positive	249
True negative	74
False positive	0
False negative	27
PPV % (CI 95%)	100 (98.17–100)
NPV % (CI 95%)	73.3 (63.86–80.97)
Accuracy % (CI 95%)	92.3 (88.97–94.68)

Legend: CI: confidence interval; PPV: positive predictive value; NPV: negative predictive value.

**Table 4:** Variable analysis for comparison between positive swabs tested by RT-qPCR and RT-LAMP according by RT-qPCR Ct and locality.

Panels of Biological Samples	*n*	RT-qPCR N1 Ct (Mean ± SD)	Positive RT-LAMP	Sensitivity % (CI 95%)
**SARS-CoV-2 RT-qPCR positive swabs**	**276**	**24 ± 4.9**	**249**	**90.2 (86.10–93.23)**
*** Swab N1 Ct ≤ 30***	**249**	**22.8 ± 5.0**	**239**	**96 (92.67–97.90)**
Rondônia	10	23.2 ± 3.7	9	90 (57.40–99.99)
Ceará	52	22.4 ± 2.8	47	90.4 (78.96–96.25)
Piauí	22	23.8 ± 2.3	22	100 (82.45–100)
Mato Grosso do Sul	101	22.5 ± 3.3	101	100 (95.6–100)
Goiás	13	21.9 ± 3.0	12	92.3 (64.5–99.99)
Rio de Janeiro	46	23.4 ± 3.86	44	95.7 (84.66–99.61)
Paraná	5	25.7 ± 3.43	4	80 (35.96–97.97)
*** Swab N1 Ct > 30***	**27**	**35.2 ± 2.34**	**10**	**37 (21.47–55.84)**
Rio de Janeiro	18	35.4 ± 2.43	5	27.8 (12.17–51.20)
Paraná	9	34.7 ± 2.21	5	55.6 (26.63–81.16)
**SARS-CoV-2 RT-qPCR positive swabs tested by MinION (Ct ≤ 30)**	**39**	**23.2 ± 3.7**	**38**	**97.4 (85.64–99.99)**

**Table 5:** RT-LAMP positivity based on the period between onset of symptoms and swab collection.

Period (Days)	*n*	RT-qPCR N1 Ct (Mean ± SD)	Positive RT-LAMP
1–5	72	22.4 ± 3.47	72
6–10	40	23.3 ± 3.99	38
>10	6	29.9 ± 6.19	3

## Data Availability

Not applicable.
